# Frontal alpha asymmetry interaction with an experimental story EEG brain-computer interface

**DOI:** 10.3389/fnhum.2022.883467

**Published:** 2022-08-12

**Authors:** Claudia Krogmeier, Brandon S. Coventry, Christos Mousas

**Affiliations:** ^1^Department of Computer Graphics Technology, Purdue University, West Lafayette, IN, United States; ^2^Department of Biomedical Engineering, University of Wisconsin−Madison, Madison, WI, United States; ^3^Wisconsin Institute for Translational Neuroengineering, University of Wisconsin-Madison, Madison, WI, United States

**Keywords:** brain-computer interfaces, electroencephalography, neurofeedback, affective computing, story, avant-garde, frontal alpha asymmetry, inter-subject correlations

## Abstract

Although interest in brain-computer interfaces (BCIs) from researchers and consumers continues to increase, many BCIs lack the complexity and imaginative properties thought to guide users toward successful brain activity modulation. We investigate the possibility of using a complex BCI by developing an experimental story environment with which users interact through cognitive thought strategies. In our system, the user's frontal alpha asymmetry (FAA) measured with electroencephalography (EEG) is linearly mapped to the color saturation of the main character in the story. We implemented a user-friendly experimental design using a comfortable EEG device and short neurofeedback (NF) training protocol. In our system, seven out of 19 participants successfully increased FAA during the course of the study, for a total of ten successful blocks out of 152. We detail our results concerning left and right prefrontal cortical activity contributions to FAA in both successful and unsuccessful story blocks. Additionally, we examine inter-subject correlations of EEG data, and self-reported questionnaire data to understand the user experience of BCI interaction. Results suggest the potential of imaginative story BCI environments for engaging users and allowing for FAA modulation. Our data suggests new research directions for BCIs investigating emotion and motivation through FAA.

## 1. Introduction

Despite the promise of brain-computer interfaces (BCIs) to create personalized and exciting experiences for users (Robinson et al., 2020), BCIs are considered “not ready” for use (Cattan, 2021), as aesthetics in BCI experiences are often not a priority, among other reasons. Thus far, BCIs are not commercially successful (Kerous et al., 2018), and present numerous challenges for researchers (Saha et al., 2021) such as simple interfaces which may not be engaging for users (Cohen et al., 2016). While there exist multiple types of BCIs, neurofeedback-based BCIs are the most popular (Kerous et al., 2018). Neurofeedback (NF) allows participants to learn to control unconscious brain activity by perceiving feedback concerning their brain activity. Feedback is often visual and simple, such as a thermometer bar (Johnston et al., 2011; Robinson et al., 2020).

While clinical NF often involves extensive training protocols for the purposes of understanding long-term psychological outcomes, BCI research harnesses NF techniques within relatively short training sessions, with the primary goal of examining novel, dynamic interaction methods for participants (Sitaram et al., 2017; Charles et al., 2020). In this study, we develop and investigate an affective BCI, which takes as input correlates of user emotion and motivation through frontal alpha asymmetry (FAA). FAA has been a target measurement in EEG NF because it is relevant to Major Depressive Disorder (MDD) (Zotev et al., 2020), and is often studied in the context of affective and motivational disorders such as MDD and anxiety. Therefore, FAA has been used as input for affective BCIs investigating novel interaction systems for emotion self-regulation. A review of the role of FAA in both emotional and motivational processes can be found at Harmon-Jones and Gable (2018).

FAA can be modulated through NF paradigms in which users learn to regulate brain activity through operant conditioning, despite a lack of volitional control (Rosenfeld et al., 1995; Aranyi et al., 2015b, 2016). Additionally, FAA can potentially serve as a biomarker to study and modulate approach motivation and affect (Briesemeister et al., 2013). In this paper, we detail our developed experimental story BCI which is designed with the goal of stimulating the imagination of the user in order to prompt novel thought content strategies for brain activity modulation. We report our experimental design and analysis, which is inspired by affective BCI work using FAA by Aranyi et al. (2016). We provide a discussion of our findings in order to inform affective BCI protocol design using complex stimuli with the broader goal of investigating user-friendly, higher complexity BCIs.

Our BCI examines participant NF interaction success within an experimental story BCI FAA NF protocol. The experimental story was designed to allow participants to exercise their imagination in order to explore mental strategies for increasing FAA. We aim to understand the efficacy of the developed BCI in allowing participants to increase their FAA score through the examination of the following research questions:

- **RQ1**: Can participants successfully engage with the developed experimental story BCI by increasing FAA?

– We hypothesize that some, but not all participants will successfully increase FAA, and that successful blocks will be characterized by large effect sizes.

- **RQ2**: Will successful blocks be characterized by an increase in left frontal cortical activity?

– We hypothesize that increased FAA will be the result of increased left frontal cortical activity rather than decreased right frontal cortical activity.

- **RQ3**: Will participant cognitive thought strategies be associated with NF interaction success?

– We hypothesize that successful participants will primarily use direct strategies while unsuccessful participants will primarily use indirect strategies to modulate brain activity.

FAA scores, measured in real-time with electroencephalography (EEG), serve as the input signal in our BCI system. For the participant, visual feedback concerning FAA is mapped to the color saturation of the main character in the story environment. Participants engaged with eight distinct story segments using imagination-based instructions for thought content strategies. Greater FAA scores increased the main character's color saturation, while lower FAA scores decreased color saturation of the main character in the dream-like story environment.

### 1.1. BCI: Frontal alpha asymmetry (FAA) and EEG

Frontal alpha asymmetry (FAA) refers to a difference in brain activity between the right and the left prefrontal cortices of the brain, and can be measured with a difference score between corresponding right and left electrode sites on an EEG device. Multiple studies have indicated that greater left relative to right prefrontal cortical activity is associated with increased approach motivation, defined as an organism's tendency to approach or expend energy in order to go toward stimuli rather than away (Harmon-Jones and Gable, 2018). Additionally, greater left relative to right prefrontal cortical activity has been associated with better emotion self-regulation ability, increased positive emotions, as well as reduced depressive symptoms (Cohen et al., 2016; Quaedflieg et al., 2016; Harmon-Jones and Gable, 2018). In affective computing, determining new ways in which humans can more directly control emotions is central to the advancement of humanity generally (Cavazza et al., 2014b), as emotional processing is diminished within numerous mental health disorders. Thus, FAA has been studied within numerous NF and BCI paradigms (Peeters et al., 2014; Aranyi et al., 2016; Mennella et al., 2017) as well as applications aiming to alleviate depression symptomology (Kelley et al., 2017).

Our objective is to investigate the efficacy of our developed BCI to facilitate FAA modulation for the user. As BCI research often emphasizes technical aspects over both human interaction and user guidance, we develop our BCI system with a focus on the cybernetic paradigm “human in the loop,” as described by Kosmyna and Lécuyer (2017). For our study, we use the Emotiv Epoc X^1^ EEG headset. This headset is minimally intrusive, quick to set up, and affordable enough to be selected by consumers; perhaps why most BCI studies incorporate a consumer grade device (Kerous et al., 2018).

### 1.2. Experimental storytelling techniques

While there is no concrete definition of “avant-garde,” there exist certain attributes of both film and game works that are considered characteristics which can often, but not always, be found within the genre (Taberham, 2018). While techniques within avant-garde films have been identified and described after their creation, we use many experimental film making techniques seen in avant-garde works within our developed experimental story BCI, seeking to use these identified techniques for the BCI experience, rather than develop a work defined as “avant-garde.” Techniques we employed include rejection of linear narrative, spatio-temporal discontinuity, lack of causal logic, and prominent stylization. The primary goal of avant-garde works is often to provide novel psychological experiences for viewers, in which viewers must exert energy and creative leaps of the imagination to think about and experience the work (Koenitz, 2017; Taberham, 2018). This exercise of the imagination is central to avant-garde works (Taberham, 2018). Therefore, our goal was to explore experimental film-making techniques identified within previously developed avant-garde works, with the goal of stimulating the participant's imagination during NF interaction. Similar to Zioga et al. (2018), we believe that interdisciplinary research such as this study will allow for creative, new approaches to understanding NF interaction in BCI experiences which may help understand these experiences in real-world settings.

By experiencing the experimental BCI story environment, participants may explore cognitive thought content strategies for brain activity modulation more freely, as creativity and imagination may be necessary to process the story environment. We investigate our assumption that an experimental story BCI may influence user mental strategies for BCI engagement positively by evaluating participant BCI engagement success through FAA measurements. We investigate commonalities in user neural engagement through inter-subject correlations (ISC) of EEG data as exploratory analyses as well, and obtain self-reported questionnaire data in order to provide a more complete picture of the user experience during BCI engagement.

## 2. Related work

Since the 1960s (Nijholt, 2015), artists have advanced the field of BCIs through the creation of applications within real-life contexts (Zioga et al., 2018). Previous research has explored multi-brain activity amongst users through art installation work (Mori, 2003; Albu, 2020), BCI collaborative control of music (Le Groux et al., 2010), and mixed media performances (Zioga et al., 2018); See Wadeson et al. (2015) for more earlier artistic BCI work. Despite many previous, creative BCIs which provide participants with imaginative, playful, as well as collaborative experiences using brain activity, BCI research investigating NF interaction success within more engaging BCI experiences is limited.

Many NF protocols investigated through user studies involve simple environments such as those which utilize status bars, single auditory tones, shape changes (Cavazza et al., 2017) or color changes (Cohen et al., 2016) for feedback concerning the user's brain activity. Moving cubes (Berger and Davelaar, 2018), histograms (Mennella et al., 2017), and box-plot meters (Quaedflieg et al., 2016) have also been used to visualize brain activity for participants. Recent work investigating the combined usage of EEG and functional magnetic resonance imaging (fMRI) NF for emotion self-regulation additionally used colored height bars to visualize two signals from EEG and two signals from fMRI (Zotev et al., 2020), as well as colored height bars in combination with pictures of positive autobiographical memories (Dehghani et al., 2020). In a study by Lackner et al. (2016), brain activity was visualized through the movement of a ball, which changed from blue to yellow with the participant's alpha band activity. Participants also saw a happy or sad smiley face after each run. Research suggests that improving the complexity of NF environments could lead to increased NF success (Cohen et al., 2016).

Cohen et al. (2016) compared EEG NF success in emotion down-regulation using a virtual animated scenario to using a simple thermometer 2D feedback system. The authors determined that the more complex, animated scenario was not only more effective for NF learning, but was also more motivating, engaging, and allowed for a greater NF learning transferability to unfamiliar environments. Berger and Davelaar (2018) examined attentional control in their BCI system, and determined that participants had a higher learning rate for increasing attention in their 3D environment than in their 2D environment. Gruzelier et al. (2010) determined greater NF learning for participants in their Cave Automatic Virtual Environment (CAVE) than for participants experiencing a screen-based rendition of the same environment. Although these studies examined varying attributes of environments with higher complexity, greater interface complexity appears to have contributed to increased NF success across different platforms.

While literature concerning affective BCIs using FAA is limited (Aranyi et al., 2015b), there exist several BCI FAA studies which utilize stories to enhance participant motivation and success through an encouragement of the imagination. Cavazza et al. (2014b) examined the use of an interactive narrative for affective interaction. In their BCI, participants were instructed to think positive thoughts in order to support the main character of their story. In their story, a female doctor experiences stressful life events such as patient death, abuse from her boss, and overworking. The participant must mentally support this character by using their thoughts in order for the doctor's outcome to improve throughout the story. Participants' brain activity was mapped to the color of the female doctor, whose color became more saturated as FAA increased. Although a proof of concept study, half of the participants were able to increase FAA with minimal training by mentally supporting the protagonist through empathetic feelings.

Aranyi et al. (2015b) developed a story-based affective BCI investigating FAA using a similar hospital story BCI environment. Participants were instructed to express angry thoughts toward an evil character, as anger has also been associated with increased approach motivation and greater FAA scores despite being a negative emotion. Brain activity was mapped to the alpha channel of the evil character. In order to prime participants to feel anger toward the evil character, this character was depicted abusing co-workers in the story environment. With greater FAA scores, the evil character became translucent, as if he was disappearing from the scene. With lower FAA scores, the evil character became more opaque, remaining in the story. Despite minimal training, participants were able to successfully engage with the BCI by increasing FAA through anger expression.

In a later study also investigating FAA, Aranyi et al. (2016) developed an affective BCI which mapped FAA scores to facial expressions of a virtual agent (character). Participants were instructed to express positive thoughts toward the agent. With increased FAA, the agent would show a positive, happy expression, and with decreased FAA, the agent would exhibit a neutral expression or look away from the participant. The authors determined that a majority of participants could successfully increase FAA through their affective BCI paradigm. For their BCI, the authors developed a short, user-friendly NF protocol designed to control for differences in FAA both *between* different NF blocks, and *across* different participants (Aranyi et al., 2016). Our methodology is largely based upon the NF protocol developed by Aranyi et al. (2016), as we seek to investigate our BCI within a user-friendly NF protocol.

We investigate BCI complexity through the development and evaluation of an experimental story environment BCI, utilizing simple visual feedback in the form of color saturation, as this visual feedback has been used in previous BCI studies. Considering that multi-component BCIs have allowed for greater NF success than single component BCIs (Jensen et al., 2013), our BCI consists of eight distinct story segments with which participants could interact through brain activity. With this study, our primary goal was to investigate an imaginative, complex BCI environment by providing participants with an experimental story experience and imagination-based instructions for engagement.

## 3. Materials and methods

### 3.1. Experimental story BCI system overview

Our BCI system was developed in the Unity game engine using the Emotiv plugin. To provide participants with real-time visual feedback of their brain activity (FAA), FAA scores were calculated by subtracting the natural log-transformed alpha power of the left electrode (F3) from the natural log-transformed alpha power of the right electrode (F4) (*ln*[*Right*] − *ln*[*Left*]) at a 2 Hz sampling rate, to match prior research by Aranyi et al. (2016). For *post-hoc* analyses, FAA scores were calculated after preprocessing the raw EEG data in EEGLAB (Delorme and Makeig, 2004). Our *post-hoc* analyses determined participant success rate in increasing FAA from *View* to *Engage* components.

Our protocol is based on that of Aranyi et al. (2016), who developed a similarly short NF protocol investigating FAA. In our protocol, participants completed eight different story blocks. Each story block consisted of a 30 s *View* component, and a 30 s *Engage* component. Each of the eight *View* and *Engage* segment pairs were separated by a 10 s *Rest* component for a total of eight *Rest* components. During *Rest*, participants were instructed to relax and try to minimize mental wandering. The timing of our *Rest* and *Prompt* components matches that of Aranyi et al. (2016). While Aranyi et al. (2016) presented *Engage* and *View* components for 40 s, our protocol differs in that we presented these components for 30 s each. This decision was made in order to both match the eight NF block protocol of Aranyi et al. (2016), while keeping the BCI experience under 15 min for participants as mentioned in Section 3.3.

During *View*, participants were instructed to count backwards. Participants counted backwards by 2 s silently starting at 500 (500, 498, 496, etc.) during each of the eight *View* components. The number by which to count backwards was determined through our pre-testing. It was determined that counting backwards by two was adequate to maintain attention while not being so difficult as to distract and frustrate participants. Counting backwards was used to control for unwanted thoughts, emotions, and mental processes (Aranyi et al., 2016).

**Figure 1:**
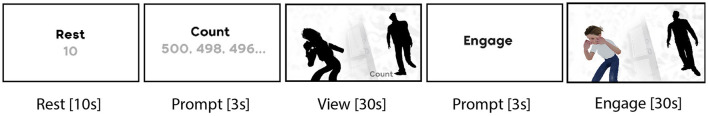
One block of our NF protocol. Components include **(A)** *Rest* [10 s], **(B,D)** *Prompt* [3 s], **(C)** *View* [30 s], and **(E)** *Engage* [30 s].

During *Engage* components, participants were instructed to use their thoughts to interact with the main character in the story, as described in more detail in the feedback mapping subsection of this paper (see Section 3.3). A 3 s prompt screen was presented to participants before both *View* and *Engage* components to indicate which component would be presented next. Participants were made aware that they would receive no visual feedback concerning their brain activity during *View* components, and would only see visual feedback during *Engage* components. Participants described mental strategies they used during the experiment on the questionnaire. One of the eight story segments of our NF protocol is shown in Figure 1.

#### 3.1.1. Story blocks

**Figure 2:**
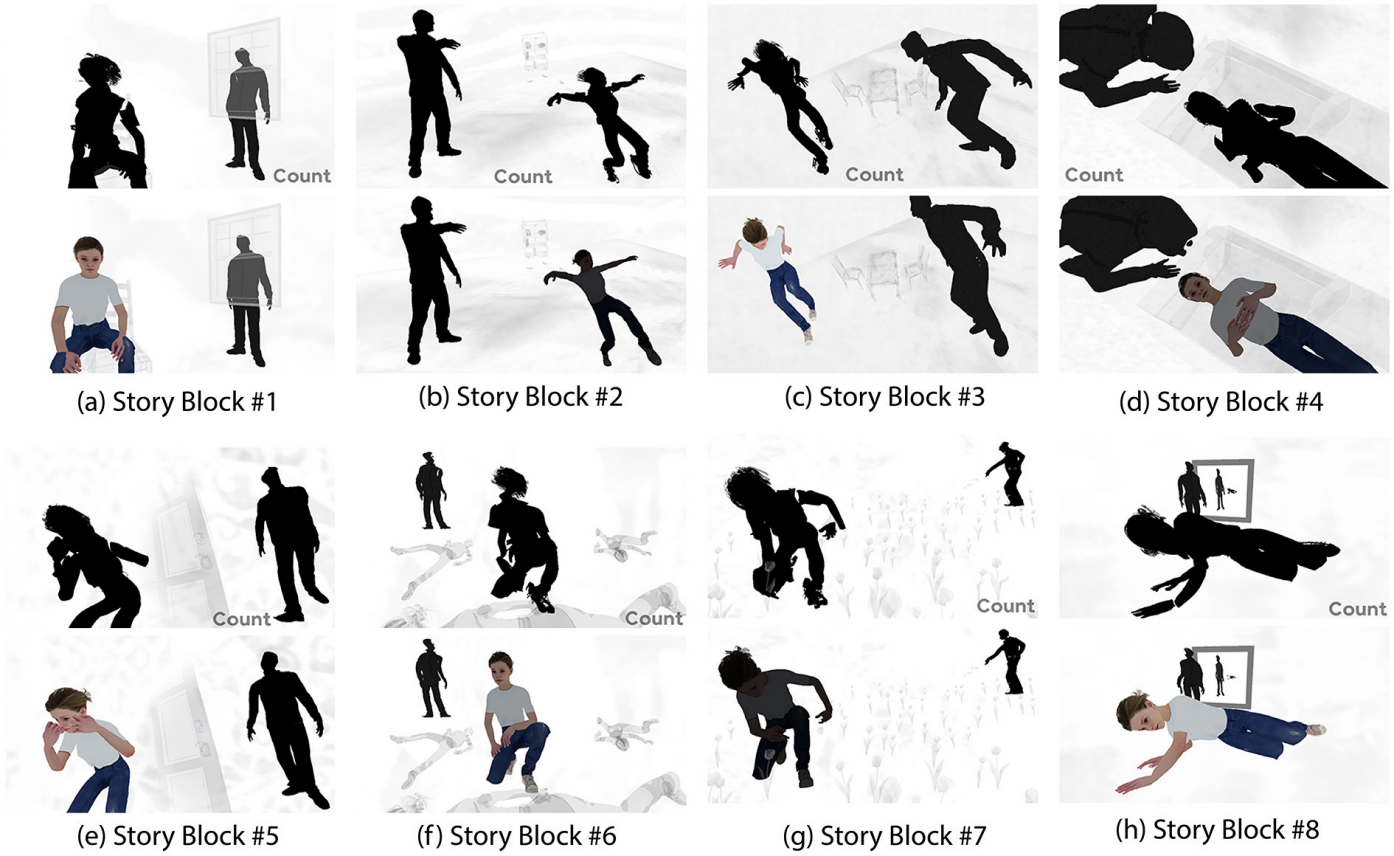
All eight story blocks, with *View* and *Engage* components. 2b and 2g demonstrate FAA which has not crossed the threshold for visual feedback, therefore, the 3d model remains dark. **(a)** Story block #1, **(b)** Story block #2, **(c)** Story block #3, **(d)** Story block #4, **(e)** Story block #5, **(f)** Story block #6, **(g)** Story block #7, **(h)** Story block #8.

We developed the story using the following design techniques: spatio-temporal discontinuity, rejection of linear narrative, abstraction of main character and narrative, and prominent stylization. Live action video footage was filmed and used for background textures within the story environment in order to create a dream-like mood. A young female 3D model was downloaded from Adobe Mixamo^2^ and used as the main character, while the background character, an adult male 3D model, was downloaded from the Microsoft Rocketbox Avatar library, MoveBox (Gonzalez-Franco et al., 2020). A shader program downloaded from the Unity asset store was applied to both characters in order to distort spatial logic concerning their forms. During *View* components, the main character and the background character were unlit (no colors), with the shader effect applied consistently throughout the *View* component. At the beginning of each *Engage* component, the main character faded out of the shader effect, returning to the original shape of the 3D model, to clearly indicate the start of an *Engage* component. During *Engage* components, participants were able to use their thoughts to increase the main character's color saturation. Characters were animated with very slow animations during both *View* and *Engage* components. Because increasing color saturation has been used previously in an FAA NF BCI (Cavazza et al., 2014b), as well as the manipulation of character transparency (Aranyi et al., 2015b), we selected an interaction mapping modality known to produce NF interaction success. In this way, we could examine the influence of an experimental story context on NF interaction success while employing a known visual feedback modality. Jensen et al. (2013) suggests examining how a combination of components might affect the user's imagination for usage in neurofeedback. All eight story blocks, consisting of both *View* and *Engage* components are shown in Figure 2.

**Figure 3:**
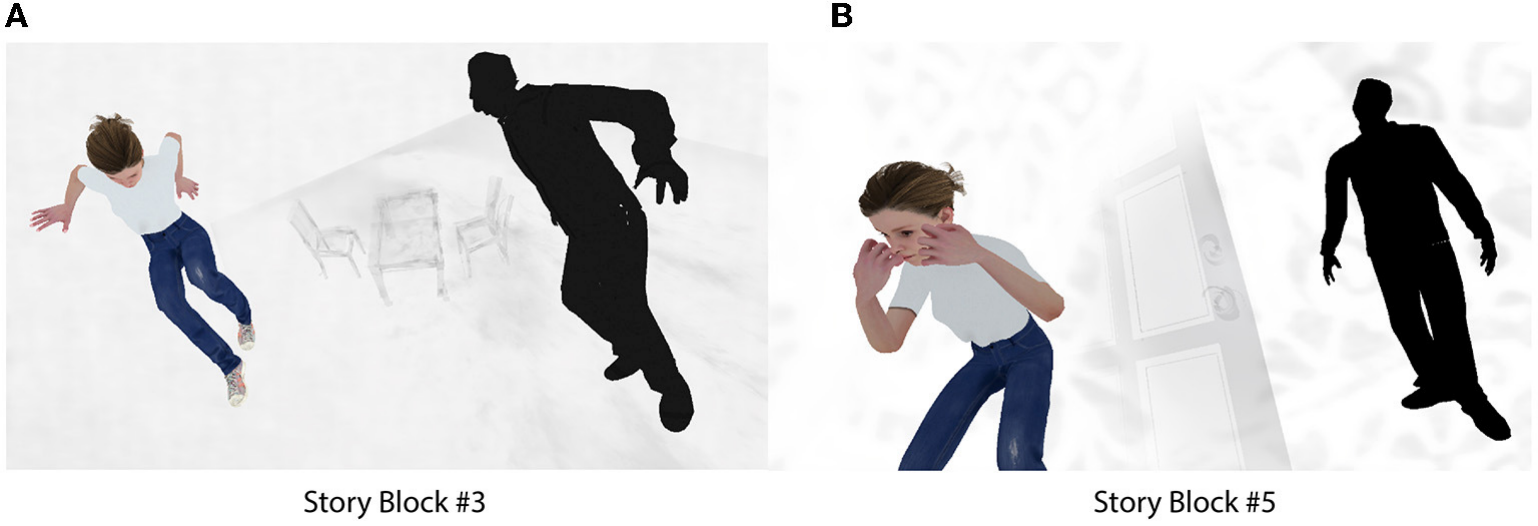
Spatio-temporal discontinuity in our story environment. **(A)** Story block #3, **(B)** Story block #5.

Concerning the spatiality of the story environment, 3D models in certain story blocks were positioned in ways that could not be possible in reality. For example, a set of chairs and a table were positioned diagonally, with a 45° tilt downwards, as the characters hovered above the environment in Story Block #3. In Story Block #5, the background character hovers well above the ground. These story blocks can be seen in Figure 3. Lacking a clear chronology of events, the story blocks were presented in the same order for all participants. Previously developed affective story BCIs have incorporated environments with negative connotations, with the goal of guiding participants more easily toward mindsets which may approach the target brain activity (Gilroy et al., 2013; Cavazza et al., 2014a,b; Aranyi et al., 2015b). Our story environment depicted the main character being symbolically watched, controlled, chased, etc., as our goal was to motivate participants to help the main character escape from the bad dream-like environment by using their thoughts. In pre-testing, participants acknowledged that the story was dream-like, open-ended, and interesting, and offered varying ideas concerning its meaning. Therefore, we concluded that the developed story environment was appropriately abstract to allow for a multiplicity of interpretations from participants.

### 3.2. BCI input

**Figure 4:**
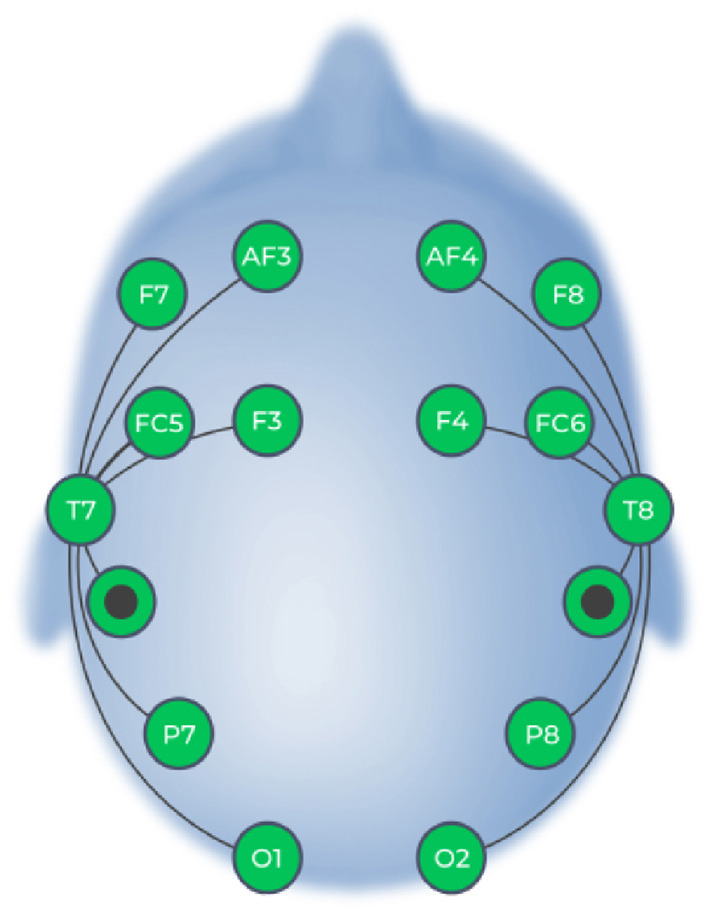
Electrodes in the Emotiv Epoc X System (image courtesy of Emotiv).

We used the Emotiv Epoc X EEG headset (Emotiv Systems Inc., San Francisco, CA, USA) to collect EEG data from participants. The Emotiv Epoc X is a consumer grade, 14 channel EEG headset which includes electrode sites AF4, AF3, F3, F4, F7, F8, FC5, FC6, O1, O2, P7, P8, T7, and T8. This electrode scheme is based on the international 10-20 system, as shown in Figure 4. Two additional electrodes served as reference and ground; the electrode located at the M1 site (Driven Right Leg [DRL]) functioned as an absolute voltage reference while the electrode located at the M2 site (Common Mode Sense [CMS]) was used for feedback noise cancellation. The electrodes are Ag/AgCl sensors which contain felt pads. Felt pads were fully soaked in saline solution prior to inserting into each electrode compartment in order to ensure the best conductance between the participant's scalp and the sensor. EEG data was recorded with a 256 Hz sampling rate and was filtered online using a built-in digital 5th order Sinc filter with a bandwidth of 16–43 Hz, with notch filters at 50 and 60 Hz.

Consumer-grade EEG data may provide researchers with more ecologically valid results, as the headset is both minimally obtrusive for participants, and has demonstrated EEG data consistent with conventional EEG recordings (Le et al., 2020). According to Gapen et al. (2016), EEG NF has made very little impact in clinical care despite being used as a clinical intervention for more than 30 years. Considering that wireless, portable and small EEG systems which can be used outside traditional laboratory environments may increase clinical relevance (Enriquez-Geppert et al., 2017), we use such an EEG device with the goal of investigating NF in a more real-world setting.

F3 and F4, along with F7 and F8, are the most commonly investigated electrode pairs in FAA research (Smith et al., 2017; Kuper et al., 2019; David et al., 2021). To maintain consistency between this study and our previous research, we selected electrode sites F3 and F4 for FAA calculation to provide participants with real-time visual feedback concerning their brain activity.

### 3.3. Participants, procedure, and feedback mapping

**Figure 5:**
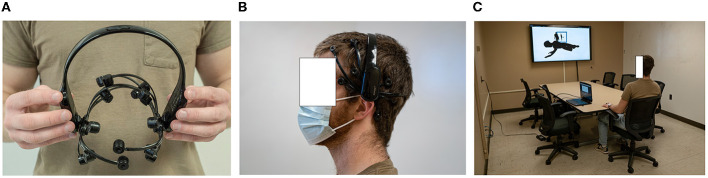
The user and devices in our BCI system. **(A)** Emotiv Epoc X EEG headset. **(B)** User wearing the headset. **(C)** The BCI room set-up.

Data was collected from 23 participants, all students at Purdue University. Due to excessive eye closure, movements, or EEG signal capture malfunctions, four participants were removed from the analysis, resulting in 19 participants (five female and 14 male; age: *M* = 19.05, *SD* = 1.02; age range: 18–22). With the exception of one participant who was left-handed, all participants were right-handed. Only one participant was currently undergoing psychiatric treatment. These participants were not identified as outliers, and were therefore included in the analysis. All participants had normal or corrected-to-normal vision. The study was approved by Purdue University's institutional review board (IRB), and participants provided written consent before participation. Similar to the BCI cinematic experience created by Pike et al. (2016), participants viewed the BCI on a large screen, in a quiet room. Participants were seated in a comfortable chair in order to minimize motion artifacts, and viewed the monitor from a comfortable viewing distance. The study was completed during the week over a three week period in late fall. Participants took 35–45 min to complete the entire study, depending on how long it took them to finish the questionnaire after the experiment. This time included EEG preparation, participant instructions, a practice round, the experiment, and questionnaire completion. The total time spent engaging with the BCI during the experiment was 13.5 min, as it has been suggested that BCI tasks longer than 15–20 min can greatly contribute to participant fatigue and, therefore, diminishing EEG data quality (Aranyi et al., 2015a). Figure 5 shows a participant in the experiment, and the devices used in the study.

The researcher first wiped the participant's lateral forehead and mastoid regions with alcohol to ensure similarly clean recordings for each participant. After the EEG headset was positioned on the head, the researcher adjusted participant hair until the Emotiv system indicated 100% EEG signal quality (all electrode indicators were green, which indicated sufficient conductance levels). To increase participant compliance with refraining from movements during the study, the researcher asked participants to try clenching their jaw, frowning, talking, and moving their limbs and face, and showed participants the noise introduced into their EEG signal during movements such as these. Additionally, the researcher showed participants what the main character would look like with full color saturation. Before the experiment, participants completed one practice block in which they practiced counting backwards during *View* components, and practiced using their thoughts to change color saturation of the main character during *Engage* components. The researcher ensured that participants clearly understood these tasks prior to starting the experiment.

Instructions concerning NF strategies are often vague so as not to constrain participants to strategies which may not be effective for them (Aranyi et al., 2016). During pre-testing, participants expressed confusion with our initial instructions. Therefore, a new set of instructions was developed. These instructions were more detailed, while maintaining an open-endedness. Defined as the ability to mentally reconstruct new information, sensations, and objects, imagination (Szczelkun, 2018) was central to our participant instructions, provided below.

*In the experiment, you will observe the girl in her dream. Your goal is to help her escape from her dream. During Engage components, you may think about new objects, sensations, ideas and possibilities, or new interpretations of the story, which may help the girl escape from this dream environment. You may imagine interacting with the girl. You may try using positive thoughts. You may explore other strategies for changing the main character's color as well*.

Following work by Aranyi et al. (2016), we defined the brain activity (FAA) threshold for visual feedback based on guidance for EEG-based FAA in NF paradigms (Rosenfeld et al., 1995). FAA values from each *View* component were used to determine the minimum and maximum FAA values necessary for visual feedback for each corresponding *Engage* component. Before calculating the minimum and maximum visual feedback threshold points, outliers that were three standard deviations higher or lower than the mean of the list of FAA values stored during *View* were removed from the list of values, in order to remove extreme values indicative of participant movements. Next, the minimum value for visual feedback during the corresponding *Engage* component was defined as the mean of the FAA values (excluding outliers) during the preceding *View* component plus 1.28 times their standard deviation. This threshold requirement for minimum values determined that visual feedback during NF would result in no feedback for 90% of the values from the *View* component, and instead show visual feedback for only the top 10% of values from the *View* component (Rosenfeld et al., 1995; Aranyi et al., 2016). The maximum value for visual feedback (the highest FAA value included in visual feedback during the *Engage* component) was set as the maximum value recorded during *View*.

**Figure 6:**
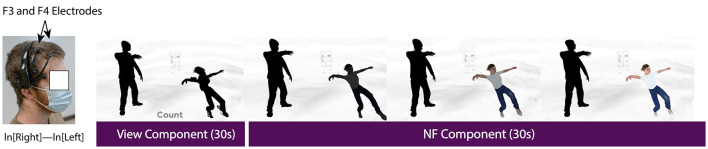
BCI system overview.

FAA values within the minimum and maximum range were linearly mapped to the main character's color saturation values, with the maximum value resulting in the main character being fully colored. Figure 6 shows an overview of our BCI system.

**Figure 7:**
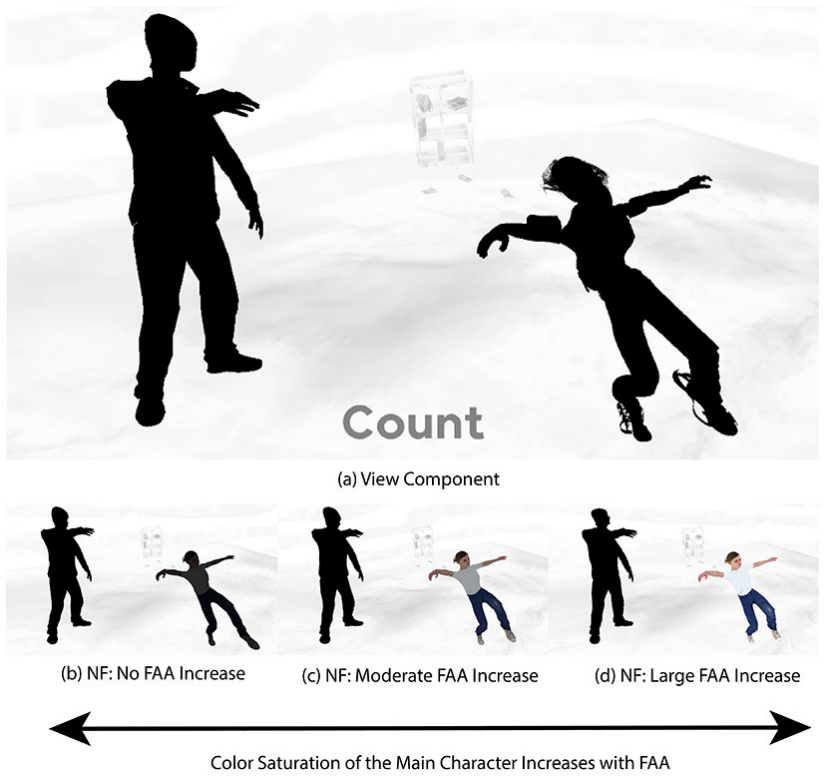
Examples of varying FAA mapped to main character color. **(a)** View component, **(b)** NF: No FAA increase, **(c)** NF: Moderate FAA increase, **(d)** NF: Large FAA increase.

*View* and *Engage* components were 30 s in length, as determined by our pre-testing. During *Engage* components only, FAA was mapped to the color saturation of the main character, thus allowing the participant to change the color saturation of the main character using their thoughts. Varying color saturation of the main character based on FAA input can be seen in Figure 7. The counting task during each *View* component provided an emotional control for each corresponding *Engage* component (Aranyi et al., 2016). A video showing the developed BCI can be found in our Supplementary material.

#### 3.3.1. EEG preprocessing and analysis

To examine participant success in increasing FAA from *View* to *Engage post-hoc*, we first preprocessed EEG data using EEGLAB (Delorme and Makeig, 2004) within MATLAB (MathWorks Inc.). Data was filtered between 1 Hz (high pass) and 30 Hz (low pass) using EEGLAB's eegfiltnew function, following FAA EEG VR research by Kisker et al. (2021). Bad channels were rejected and interpolated. After applying an average reference, Independent Component Analysis (ICA) was run on the data, and eye components with 50% probability were removed. EEGLAB's spectopo function was used on the cleaned data to calculate the power spectra in the 8–13 Hz alpha range. We subtracted the natural log-transformed alpha power of the left electrode (F3) from the natural log-transformed alpha power of the right electrode (F4) to calculate FAA: (*ln*[*Right*]−*ln*[*Left*]) (Smith et al., 2017). FAA was calculated for each *View* and *Engage* component for each participant. Within each 30 s component, FAA was calculated from 2 s segments to create a distribution of FAA scores.

#### 3.3.2. FAA and alpha power measurements

We examine both FAA measurements as well as alpha power measurements from the left and right electrode sites individually in our study. FAA is calculated by subtracting the natural log of the alpha power from the left electrode site from the natural log of the alpha power of the right electrode site. However, this FAA score does not provide information concerning how much the alpha power from either the left or right electrode site contributed to the FAA score (Smith et al., 2017). Like Aranyi et al. (2016), we wanted to understand the contribution of right and left prefrontal cortical activity to successful FAA scores, as a higher FAA score could be indicative of either increased left cortical activity or decreased right cortical activity (Smith et al., 2017). Therefore, we conducted additional analyses to understand how alpha power on the right and left sides changed from *View* to *Engage* blocks. In this way, we wanted to understand if FAA which increased from *View* to *Engage* did so because of increased left prefrontal cortical activity, or decreased right prefrontal cortical activity. Because alpha power is inversely related to cortical activity (Allen et al., 2004), a higher alpha power from the right electrode would indicate lower cortical activity from the right electrode. Results concerning alpha power from the right and left hemispheres are described in Sections 4.1 and 4.2.

#### 3.3.3. Inter-subject correlations (ISC) analysis

To determine between subject reliability of evoked responses, inter-subject correlations (ISC) were calculated (adapted from Dmochowski et al., 2012; Cohen et al., 2016). Briefly, sample electrode covariances were calculated as:


(1) $$\begin{array}{l} R_{ij}=\frac{1}{N}\underset{t}{\sum }x_{i}\left(t\right)x_{j}\left(t\right), \end{array}$$

where *x*_*n*_ represents the time series electrical activity recorded on all electrodes of subject *n*. Within and between subject correlations were then calculated as:


(2) $$\begin{array}{l} R_{w}=\frac{1}{N}\underset{n}{\sum }R_{nn} \end{array}$$

(3) $$\begin{array}{l} R_{b}=\frac{1}{N\left(N-1\right)}\underset{i}{\sum }\underset{j,j\neq i}{\sum }\underset{k}{\sum }\left(x_{i}\left(t\right)-{\bar{x}}_{i}\right){\left(x_{k}\left(t\right)-{\bar{x}}_{k}\right)}^{T}, \end{array}$$

where *x*_*n*_(*t*) is the measured scalp voltage on channel *n* and ${\bar{x}}_{n}$ is the time average of channel *n*. The value *R*_*b*_ represents the summation over all cross-covariances of all electrodes of all subjects. Maximal covariances are then calculated as component projections:


(4) $$\begin{array}{l} Component_{i}=\frac{v_{i}^{T}R_{b}v_{i}}{v_{i}^{T}R_{w}v_{i}}, \end{array}$$

where *v*_*i*_ is the *i*^*th*^ eigenvector of the matrix $R_{w}^{-1}R_{b}$. Intersubject correlation is then calculated as:


(5) $$\begin{array}{l} ISC=\underset{i}{\sum }C_{i}. \end{array}$$

In keeping with previous ISC studies (Dmochowski et al., 2012; Cohen et al., 2016), the three largest correlated components are utilized in calculating ISC. Time resolved correlations were formed by calculating ISCs across all electrodes for all subjects for each scene within 1 s windows with an 800 ms overlap between windows.

Spatial distributions of maximal correlation coefficients were calculated using “forward model” analyses (Parra et al., 2005), specifically:


(6) $$\begin{array}{l} A=R_{w}W{\left(W^{T}R_{w}W\right)}^{*}, \end{array}$$

where *W* is the set of linear spatial filters:


(7) $$\begin{array}{l} w_{ij}=argmax_{w}\frac{w^{T}R_{ij}w}{\sqrt{w^{T}R_{ii}w}\sqrt{w^{T}R_{jj}w}}, \end{array}$$

and ^*^ designates the Moore-Penrose pseudo-inverse (Ben-Israel and Greville, 2003), a generalization of the matrix inverse which calculates a least-squares best fit *via* singular value decomposition to calculate a matrix inverse. The Moore-Pensore is used to ensure numerical stability between electrodes with varying levels of activation.

Statistical significance of ISCs was assessed *via* permutation testing (Fischer, 1971). Electrode time series were randomly shuffled in time and empirical p-values reflective of one-tailed type-1 error in rejecting the null hypothesis were calculated from the distribution of time-shuffled correlations after 1,000 iterations. A window of ISC was considered significant if *p* < 0.05 after multiple comparisons correction using the Benjamini-Hochberg procedure to control for the false discovery rate (Benjamini and Hochberg, 1995).

## 4. Results and discussion

**Figure 8:**
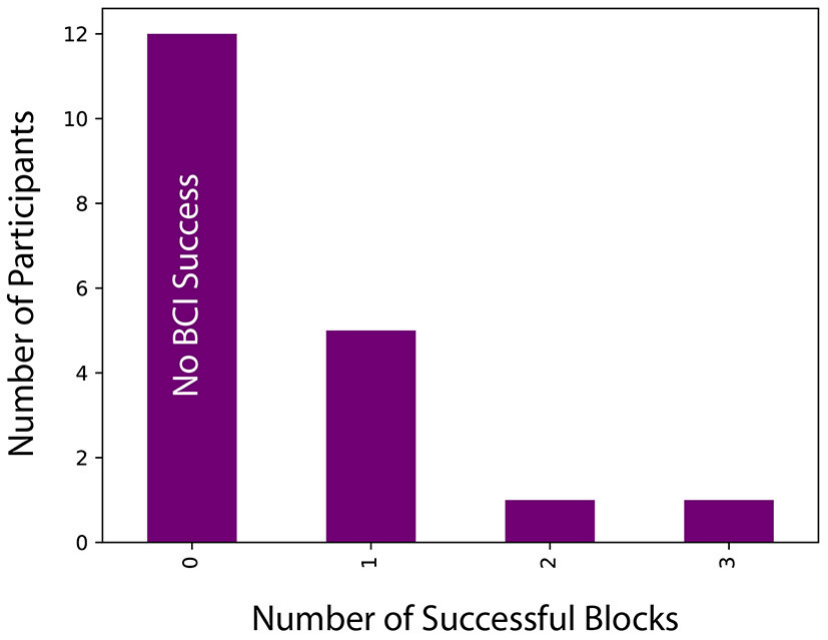
Distribution of successful blocks across participants.

One hundred and fifty-two blocks were identified for analysis (eight blocks for each of the 19 participants). Block success was defined as a statistically significant increase in average FAA during *Engage* components compared to average FAA during *View* components within the same block. For example, we compared the *Engage* and *View* components for Story Block #1, then for Story Block #2, and so on, for each participant individually. Block success was determined using paired samples *t*-tests. Data was inspected using Q-Q plots of the residuals and was normally distributed. Like Le et al. (2020), who also investigated FAA using an Emotiv EEG recording device, we winsorized outliers in the data in order to reduce the influence of extreme scores. We winsorized the data by replacing outlier values with the next minimum or maximum value for that component (as the data included both positive and negative FAA values). Seven out of 19 participants (37%) achieved statistically significant NF success in increasing their FAA score in at least one of the eight blocks, for a total of 10 successful blocks in the experiment. Figure 8 shows the distributions of successful blocks across participants.

**Figure 9:**
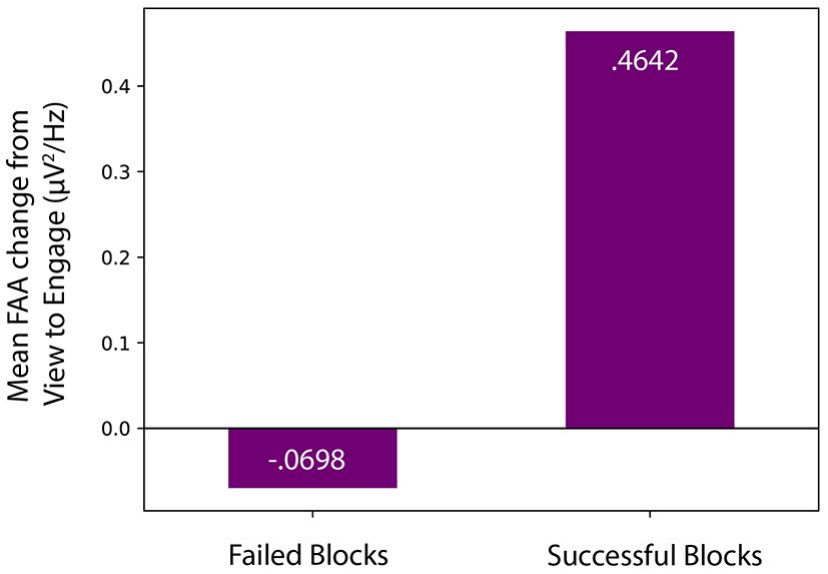
Mean FAA change from *View* to *Engage* for both successful and unsuccessful blocks.

We investigated effect sizes for successful blocks, in which average FAA during *Engage* was statistically significantly greater than average FAA during *View*. Effect sizes for successful participant blocks were calculated using Hedge's g using a standard deviation of the difference. The smallest and largest effect sizes detected were 0.477 and 1.27, respectively, with an average effect size of 0.749. Similar to work by Aranyi et al. (2016), our effect size values were negatively skewed, indicative of larger effect sizes for successful blocks. Additionally, we determined that successful blocks were characterized by a large FAA increase (*M* = 0.46, *SD* = 0.04) from *View* to *Engage*, while unsuccessful blocks were characterized by a very small decrease in FAA (*M* = −0.07, *SD* = 0.01), as shown in Figure 9. Despite few successful blocks, our **RQ1** is supported as some participants successfully increased FAA scores from *View* to *Engage*, and successful blocks were characterized by large effect sizes as well as large FAA increases.

### 4.1. Successful blocks characterized by increased left prefrontal cortical activity from *View* to *Engage*

We used a two-way repeated measures analysis of variance (ANOVA) with average alpha power as the dependent variable, and *Component Type* (*View* or *Engage*) and *Side* (*Left* [F3] or *Right* [F4]) as factors in the analysis. Alpha power which was not normally distributed was transformed following recommendations by Templeton (2011) prior to conducting our parametric analyses. Our simple main effects analysis used Bonferroni confidence interval adjustment.

We found a statistically significant interaction effect between *Side* and *Component Type* [Λ = 0.561, *F*_(1,9)_ = 7.035, *p* = 0.026, $\eta_{p}^{2}=0.439]$. Therefore, we conducted simple main effects analyses for both *Side* and *Component Type*. We determined a simple main effect for *Side*, finding that *Left* alpha power (*M* = 8.74, *SD* = 9.41) during *View* was significantly greater than *Right* alpha power (*M* = 3.90, *SD* = 3.45) during *View* [Λ = 0.619, *F*_(1,9)_ = 5.548, *p* = 0.043, $\eta_{p}^{2}=0.381]$. However, *Left* alpha power (*M* = 2.34, *SD* = 1.49) did not significantly differ from *Right* alpha power (*M* = 2.51, *SD* = 1.29) during *Engage* [Λ = 0.960, *F*_(1,9)_ = 0.373, *p* = 0.556, $\eta_{p}^{2}=0.040]$.

We also found a simple main effect for *Component Type*, determining that *Left* alpha power (*M* = 8.74, *SD* = 9.41) during *View* was significantly greater than *Left* alpha power (*M* = 2.34, *SD* = 1.49) during *Engage* [Λ = 0.591, *F*_(1,9)_ = 6.231, *p* = 0.034, $\eta_{p}^{2}=0.409]$. However, we found that *Right* alpha power (*M* = 3.90, *SD* = 3.45) during *View* did not significantly differ from *Right* alpha power (*M* = 2.51, *SD* = 1.29) during *Engage* [Λ = 0.745, *F*_(1,9)_ = 3.085, *p* = 0.113, $\eta_{p}^{2}=0.255]$.

**Figure 10:**
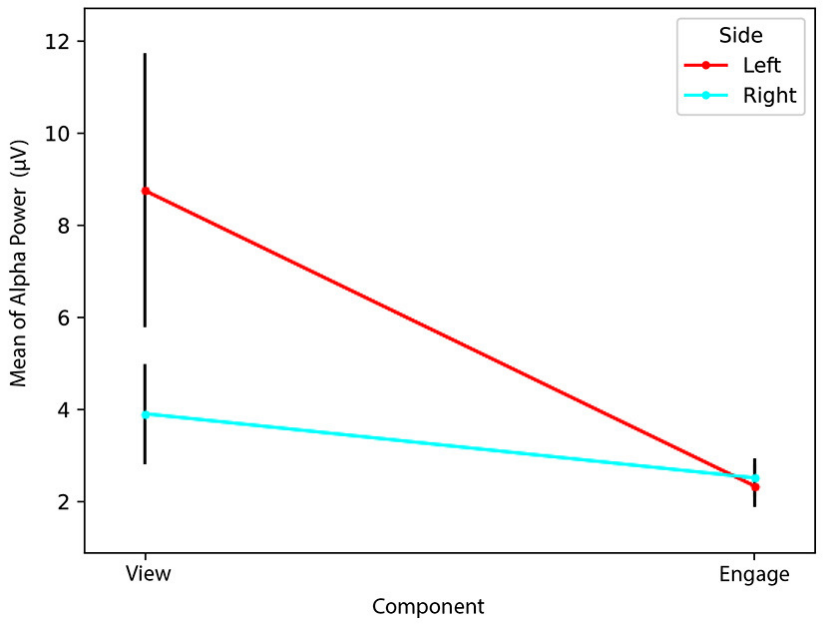
Unsuccessful Blocks. Both *Right* and *Left* alpha power decrease from *View* to *Engage*, although *Left* to a greater (significant) extent.

Our results indicate greater *Right* cortical activity than *Left* cortical activity during *View*. Because greater relative *Right* than *Left* cortical activity would indicate a lower FAA than if *Left* > *Right*, it is possible that successful blocks were characterized by a lower starting FAA during *View* compared to *Engage*. Although *Right* cortical activity was greater than *Left* cortical activity during *View, Right* and *Left* cortical activity do not differ significantly during *Engage*, as seen in Figure 10. Considering the decrease in alpha power (increase in cortical activity) on the *Left* from *View* to *Engage*, an increase in *Left* cortical activity appears to have contributed to increased FAA, rather than a decrease in *Right* cortical activity from *View* to *Engage*. We consider our **RQ2** partially supported, as an increase in *Left* cortical activity from *View* to *Engage* contributed to increased FAA during *Engage*, despite determining no difference between *Left* and *Right* cortical activity during *Engage*. Unlike Aranyi et al. (2016), our results indicate a lateralization (*Left* > *Right* alpha power) during *View*, but no lateralization during *Engage*.

Considering the increase in cortical activity on both *Left* and *Right* sides from *View* to *Engage* (although, not a statistically significant increase on the *Right* side), our results suggest an increase in mental effort from *View* to *Engage*. This is in line with our self-reported data examining the mental demand of both components. Participants used a Likert scale taken from the NASA Task Load Index (NASA TLX) (Hart, 2006), an assessment of mental workload, to rate mental effort necessary to perform both *View* and *Engage* tasks. We conducted a one-tailed paired samples *t*-test [*t*_(18)_ = −3.366, *p* = 0.002] and determined that *Engage* mental demand (*M* = 4.74, *SD* = 1.59) was significantly greater than *View* mental demand (*M* = 2.63, *SD* = 1.83).

### 4.2. Unsuccessful blocks characterized by comparable increases in *Right* and *Left* prefrontal cortical activity

Of the 142 unsuccessful blocks, 6% were characterized by a decrease in FAA. Therefore, unsuccessful blocks were largely characterized by no change in FAA from *View* to *Engage*. We investigated the contribution of *Left* and *Right* alpha power in unsuccessful blocks as well, and determined a statistically significant interaction between *Side* and *Component Type* [Λ = 0.896, *F*_(1,141)_ = 16.428, *p* < 0.001, $\eta_{p}^{2}=0.104]$. Simple main effects analysis of *Side* was also significant [Λ = 0.795, *F*_(1,141)_ = 36.467, *p* < 0.001, $\eta_{p}^{2}=0.205]$, indicating that *Left* alpha power (*M* = 2.00, *SD* = 1.72) was significantly lower than *Right* alpha power (*M* = 2.68, *SD* = 2.03) during *View*. This is the opposite of our results for successful blocks, in which *Left* alpha power was significantly greater than *Right* alpha power during *View*. It appears that unsuccessful blocks may have instead been characterized by a higher starting FAA during *View*, and therefore, it may have been significantly more difficult to increase FAA further during *Engage*.

**Figure 11:**
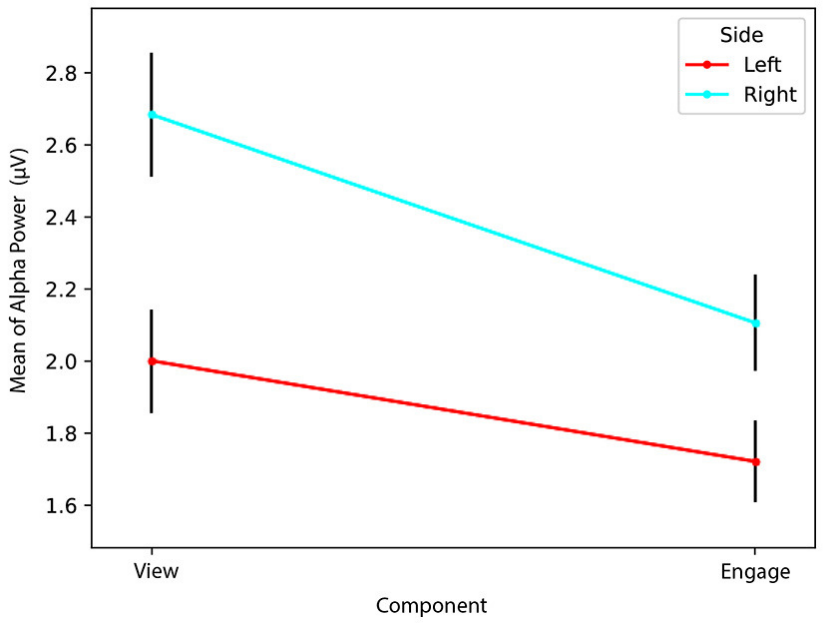
Successful Blocks. Both *Right* and *Left* alpha power decrease from *View* to *Engage*, but *Right* remains significantly greater than *Left* during *Engage*.

Unlike with successful blocks, we additionally determined a statistically significant difference [Λ = 0.873, *F*_(1,141)_ = 20.460, *p* < 0.001, $\eta_{p}^{2}=0.127]$ between *Left* (*M* = 1.72, *SD* = 1.37) and *Right* alpha power (*M* = 2.11, *SD* = 1.57) during *Engage*, indicative of greater *Left* than *Right* cortical activity during *Engage* for unsuccessful blocks. Our results indicate that the lack of a significant increase in FAA from *View* to *Engage* found in unsuccessful blocks may have been influenced by the greater increase in *Right* than *Left* cortical activity from *View* to *Engage*, as seen in Figure 11.

A simple main effect analysis of *Component Type* was also statistically significant [Λ = 0.769, *F*_(1,141)_ = 42.252, *p* < 0.001, $\eta_{p}^{2}=0.231]$. We determined that *Right* alpha power during *View* (*M* = 2.68, *SD* = 2.03) was significantly greater than *Right* alpha power during *Engage* (*M* = 2.11, *SD* = 1.57), indicative of greater increased *Right* cortical activity during *Engage* than during *View*. *Left* alpha power during *View* (*M* = 2.00, *SD* = 1.72) was significantly greater than *Left* alpha power during *Engage* (*M* = 1.72, *SD* = 1.37), indicative of greater *Left* cortical activity during *Engage* than during *View* [Λ = 0.900, *F*_(1,141)_ = 15.736, *p* < 0.001, $\eta_{p}^{2}=0.100]$. Therefore, self-reported data concerning mental demand of *View* and *Engage* blocks is in line with unsuccessful blocks as well, in that more cortical processing took place during *Engage* on both *Right* and *Left* sides.

Results suggest that while successful blocks were characterized by a relatively greater increase in *Left* compared to *Right* cortical activity, unsuccessful blocks were characterized by a comparable increase in both *Right* and *Left* cortical activity. While successful blocks may have also been characterized by a relatively lower starting FAA during *View*, making an increase in FAA more feasible during *Engage*, unsuccessful blocks may have been characterized by a higher starting FAA during *View*, perhaps making an increase in FAA during *Engage* more difficult. Considering that there was also greater cortical activity on both sides during *Engage for unsuccessful blocks*, it is possible that participants exerted greater mental effort during unsuccessful blocks as alpha power is thought to be inversely related to cortical network activity (Allen et al., 2004; Smith et al., 2017). Mean *View* and *Engage* FAA values for each block for each participant are shown in Table 1.

**Table 1:** The mean FAA values of the distribution of 15 FAA values, for each *View* and *Engage* Component of each story block, for each participant.

	**Story Block #1**		**Story Block #2**		**Story Block #3**		**Story Block #4**		**Story Block #5**		**Story Block #6**		**Story Block #7**		**Story Block #8**	
**ID**	**View**	**Engage**	**View**	**Engage**	**View**	**Engage**	**View**	**Engage**	**View**	**Engage**	**View**	**Engage**	**View**	**Engage**	**View**	**Engage**
#1	0.115	−0.267	−0.041	−0.182	-0.125	0.003	−0.098	0.172	0.188	0.058	−0.031	0.018	0.120	0.198	0.029	−0.077
#2	−0.193	0.057	0.150	0.068	−0.012	0.114	0.092	0.290	0.035	0.202	0.186	0.232	0.058	0.089	0.070	0.017
#3	−0.511	−0.114	−0.626	−0.535	−0.576	−0.595	−0.825	−0.374	−0.560	−0.484	−0.434	−0.563	−0.473	0.067	−0.173	−0.548
#4	0.416	0.484	0.415	0.587	0.812	0.202	0.119	0.464	0.400	0.615	0.508	0.239	0.091	0.291	0.588	0.684
#5	−0.610	−1.040	−0.872	−1.070	−0.944	−1.099	−1.058	−0.847	−0.996	−0.608	−0.815	−0.993	−0.981	−0.561	−-0.894	−1.101
#6	0.908	1.408	0.977	1.055	0.853	1.221	1.090	0.697	1.084	0.927	0.911	0.787	0.872	0.924	1.059	1.000
#7	0.237	0.339	0.496	0.326	0.063	0.557	0.353	0.212	0.271	0.251	0.205	0.032	0.349	0.471	0.352	0.095
#8	−0.497	−0.406	−0.020	−0.447	−0.065	−0.478	−0.340	−0.468	−0.134	−0.272	−0.269	−0.273	−0.231	−0.448	−0.216	−0.235
#9	0.493	0.632	0.339	0.408	0.371	0.458	0.644	0.560	0.454	0.473	0.513	0.570	0.592	0.478	0.423	0.629
#10	0.667	0.271	0.690	0.207	0.576	0.408	0.611	0.465	0.485	0.288	0.663	0.504	0.570	0.522	0.687	0.287
#11	−0.105	−0.104	−0.048	0.176	0.206	0.198	0.423	0.089	0.185	0.177	0.623	0.044	−0.096	0.086	0.503	−0.077
#12	0.645	0.396	0.696	0.576	0.593	1.057	0.762	0.482	0.911	0.814	0.387	0.481	0.673	0.742	0.618	0.423
#13	0.038	−0.268	−0.582	−0.087	−0.122	0.257	0.223	0.114	−.684	0.051	0.064	−0.203	0.053	−0.107	−0.446	−0.163
#14	0.635	0.826	0.475	0.649	0.677	0.840	0.839	0.777	0.561	0.509	0.602	0.810	0.695	0.528	0.573	0.759
#15	1.070	0.701	1.216	0.474	0.950	0.891	1.161	0.852	1.065	0.882	0.858	0.982	0.878	1.179	1.138	0.656
#16	−0.048	0.218	0.248	0.190	0.141	0.105	0.545	0.494	0.116	0.142	−0.132	0.462	0.232	0.427	0.154	0.291
#17	1.076	0.776	0.988	0.632	0.851	0.797	0.595	0.679	0.818	0.515	0.602	0.597	0.704	0.726	0.747	0.630
#18	−0.039	−0.218	0.294	0.176	−0.033	0.442	0.291	0.281	0.428	0.269	0.636	0.236	−0.200	0.059	0.330	−0.006
#19	0.606	0.228	0.700	0.402	0.788	0.332	0.438	0.321	0.105	0.412	0.499	0.251	0.417	0.426	0.599	0.326

### 4.3. Inter-subject correlations (ISC) suggest varying strategies for achieving NF success

ISCs and EEG forward modeling were calculated to explore spatial distributions of sources of neural activity and to assess the between subject reliability of evoked responses during *View* and *Engage* blocks for each story block. As subject response to visual feedback is not a stationary process, ISCs were calculated in 1 s windows across all subjects for each story block and decomposed into the three largest correlation components. Percentages of windows which were significant above chance were calculated and reported in Table 2. Components during *View* showed a larger percentage of significant windowed correlation with the exception of story blocks #2, #6, and #7, which showed an equal or larger percentage of significant windows during *Engage*. Results suggest that subject neural activity was significantly less correlated during *Engage*, as neural activity showed longer durations of significant correlated activity during *View*, with the exception of story blocks #2, #6, and #7. Longer de-correlated activity during *Engage* could be due to different thought content strategies for changing the main character's color saturation employed by different subjects. Higher correlations during *View* are not unexpected as subject counting strategies are likely more consistent than strategies taken during the NF task.

**Table 2:** Percentage of windows which show significant intersubject correlation for each *View* and *Engage* component of each story block for the three largest correlation component projections.

Percentage Significant ISC Windows																
Component	**Story Block #1**		**Story Block #2**		**Story Block #3**		**Story Block #4**		**Story Block #5**		**Story Block #6**		**Story Block #7**		**Story Block #8**	
	**View**	**Engage**	**View**	**Engage**	**View**	**Engage**	**View**	**Engage**	**View**	**Engage**	**View**	**Engage**	**View**	**Engage**	**View**	**Engage**
#1	13.51	10.81	5.41	9.46	14.86	9.46	21.62	16.89	14.19	9.46	6.76	22.97	8.78	8.78	11.49	5.41
#2	15.54	8.78	1.35	11.49	22.97	8.78	2.70	.00	10.14	14.19	7.43	10.81	6.76	14.19	12.16	6.08
#3	9.46	8.78	4.73	4.05	9.46	6.08	8.78	4.73	6.08	10.14	5.41	9.46	8.11	9.46	4.76	6.76

**Figure 12:**
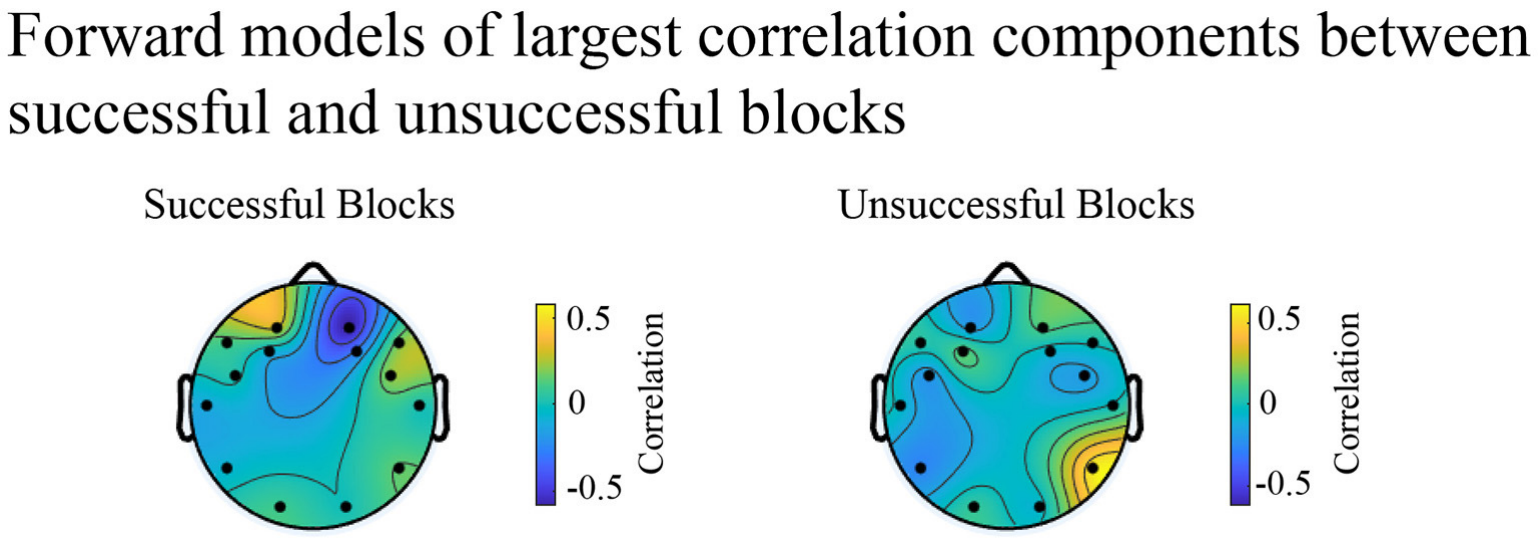
Spatial distribution of between subject correlations shows no consistent pattern of neural activity across story blocks in engage and view states.

Blocks were split into successful and unsuccessful groups containing all successful or unsuccessful blocks respectively. Forward EEG models of ISC of alpha power quantifying largest correlation components across all subjects were calculated for each group. As expected, forward models verify that successful blocks show increased left-frontal activity relative to right-frontal activity compared to unsuccessful blocks (Figure 12). These data suggest that subject success on a given block was marked by robust and reliable asymmetric activity.

### 4.4. Self-reported data

We collected self-reported data from our questionnaire to inform us of the human experience of brain activity modulation with the experimental BCI story environment. In this section, we present our results concerning thought content strategies for brain activity modulation, perceived success in BCI engagement, and levels of enjoyment and frustration.

#### 4.4.1. Participant strategies

All participants were asked to describe their strategies during *Engage* blocks. We first investigated *Direct* and *Indirect* strategies during *Engage* (Aranyi et al., 2016). We defined *Direct* strategies as those which directly involved interacting with the main character or the story environment. Examples of *Direct* strategies include “*protecting [the main character] and making her feel safe*,” “*speak[ing] with her using my thoughts*,” and “*creating exit strategies as well as trying to comfort or help the girl escape*.” *Indirect* strategies instead involved thoughts that were unrelated to the story environment, such as recalling past experiences and memories. Examples of *Indirect* strategies include thinking of “*something that makes me happy, like the color purple… or my friends*,” and “*[having] a conversation with myself*.” We determined that seven participants used *Indirect* strategies while 12 participants used *Direct* strategies. We used a Chi-Squared test and determined that there was no significant association between block success (Success/Failure) and strategy (*Direct*/*Indirect*), *X*^2^(1, *N* = 19) = 0.833, *p* = 0.361, Cramer's *V* = 0.209. In future work, it would be necessary to control for strategy type in order to make conclusions about any relationship between strategy and NF interaction success.

Our data shows five out of seven successful participants used *Direct* strategies, while six out of 12 unsuccessful participants used *Direct* strategies. Similarly, Aranyi et al. (2016) determined that *Indirect* strategies were more prominent for unsuccessful participants. Although block success was not significantly associated with strategy type, our **RQ3** is partially supported, as a majority of successful participants used *Direct* strategies during *Engage*, while half of unsuccessful participants used *Indirect* strategies.

#### 4.4.2. Perceived success, enjoyment, frustration

Participants also provided subjective ratings of their success during *Engage* blocks using an 8-point Likert scale. We used a Pearson Bivariate one-tailed correlation and determined no statistically significant correlation between perceived success and number of successful blocks *r*_(19)_ = −0.089, *p* = 0.358, suggesting that participants were unclear about NF success. However, enjoyment (*M* = 5.89, *SD* = 1.66) was significantly greater than frustration (*M* = 2.79, *SD* = 2.28), as determined by a one-tailed paired *t*-test [*t*_(18)_ = 4.083, *p* < 0.001].

## 5. Conclusion

Our results indicate that seven out of 19 participants were able to use thought strategies to modulate brain activity (FAA) when engaging with the complex story-based BCI environment. Results suggest that successful *Engage* blocks are characterized by both a large increase in FAA from *View* to *Engage*, and a large effect size, while unsuccessful blocks are characterized by relatively no change in FAA from *View* to *Engage*. Considering both successful and unsuccessful blocks, prefrontal cortical activity on left and right sides was significantly greater during *Engage* than *View*, in line with self-reported data which demonstrates participants found *Engage* blocks significantly more mentally demanding than *View* blocks. With our ISC analysis, we determined that components during *View* showed a larger percentage of significant correlation time than components during *Engage*. Therefore, it is likely that patterns of participants' neural activity were more similar during *View*, in which participants counted, than during *Engage*, in which thought content strategies were relatively unconfined. During narrative film viewing, attention has been shown to modulate similar EEG evoked responses (Cohen et al., 2017). Therefore, it is possible that participants differed significantly in their ability to pay attention during *Engage* blocks.

Our investigation of left and right prefrontal cortical activities during successful blocks suggests that an increase in left cortical activity, rather than a decrease in right cortical activity from *View* to *Engage* was influential in increased FAA scores from *View* to *Engage*, which suggests successful blocks may have been the result of increased approach motivation. Data suggests increasing right *and* left prefrontal cortical activity from *View* to *Engage* contributed to the unchanging FAA score for unsuccessful blocks. Forward models of decomposed electrical activity of strongest correlated responses also confirmed that successful blocks were marked with robust left-right asymmetries. It is possible that blocks in the unsuccessful category have some level of asymmetry, but at lower levels than successful blocks. As such, future work in FAA based BCIs should titrate the level of left-right asymmetric activity with reliable FAA detection and controlability.

While our results indicate multiple successful blocks across ~37% of participants, the percentage of unsuccessful participants (63%) in this study is higher than typical BCI illiteracy rates at 15–30% (Saha et al., 2021). However, BCI illiteracy, when participants cannot modulate brain activity within the time frame of the study, is not well-understood. We offer several ideas concerning the difference in successful blocks between our study and that of Aranyi et al. (2016). First, our consumer-grade EEG device was likely not sensitive enough to detect small changes in FAA, as indicated by successful blocks characterized by moderate to large effect sizes only. Second, our self-reported results concerning perceived success indicate that participants did not understand when they were successful in modulating brain activity, which may arise as a diminished neural response ISC seen in *Engage* compared to *View* data. Because success was unclear, participants may not have felt rewarded by visual feedback of their brain activity. Reward is essential for learning through operant conditioning, and may explain some of the lack of BCI engagement success.

Considering work by Hasson et al. (2008), it is possible that certain film works may elicit more similar, homogeneous brain responses across viewers, as seen through higher ISCs during certain films over others. In their study, the authors determined that higher ISCs were found when participants viewed a film directed by an accomplished film director, compared to unstructured video recorded in a park environment. Hasson et al. (2008) conclude that films which can better guide attention may lead to greater ISCs. Additionally, it is known that films played backwards do not have as high ISCs as those played in their original temporal order (forward, typically; Hasson et al., 2008), or when scenes are scrambled in time (Dmochowski et al., 2012). Because the experimental story presented was designed to be abstract, unguided, and open-ended to interpretation, our limited ISCs results are expected. Additionally, it is likely that the open-ended nature of the experimental narrative contributed to the comparable ISCs during View and Engage blocks as well, as the unguided nature of the story blocks led to higher variances in brain activity than might have been found with story blocks with a more concrete, guided story. While a more guided story BCI may have elicited greater ISC, future research is needed to determine how ISC relates to NF interaction success with different kinds of BCI experiences.

Previous research has explored participants' prior BCI experience, as it “could possibly influence [the participant's] participation in the study” (Zioga et al., 2018; p. 4). Considering that none of the participants had previously interacted with a BCI, it is possible that participants expected a different, perhaps mentally passive experience, in which brain activity was recorded “as is,” without the effort necessary to engage with NF, such as the BCI experience of Zioga et al. (2018). Although our goal was to provide participants with open-ended ideas for NF interaction, story blocks may not have been salient enough for participants to achieve high levels of NF success. While some participants expanded on the experimental story narrative in creative ways through self-reported data, others were unable to find any meaning or narrative structure within what one participant referred to as “moving images.” Questionnaire data suggests that participants did not apply one strategy consistently throughout *Engage* blocks, as they did in the case of Aranyi et al. (2016). Additional research concerning strategy consistency in BCI engagement would elucidate our findings. Similar to Aranyi et al. (2016), we did not control for valence (positive of negative affect) of NF strategies. Questionnaire data showed that participants used sad as well as happy thought content strategies during *Engage*, which likely influenced NF success rates. According to Friedrich et al. (2014) however, participants can be divided into three groups: one third of participants who gain control of brain signals immediately, one third who cannot gain control of brain signals, and *one third who can only gain control of brain signals after training*. With this in mind, it is possible that our system did not allow for adequate training for individuals who needed training to be successful, as only one third of the participants achieved NF interaction success in our study. Research has shown that NF success can be driven by differences in learner (participant) strategies. For example, Davelaar et al. (2018) found that learners (participants who could gain control of their brain activity) described feeling, or being aware of something, while non-learners (participants who did not gain control of their brain activity during the course of the training session) exerted significant mental effort or tried to maintain strict attentional focus. Davelaar et al. (2018) describe these strategies on a feeling-sensing continuum, where NF success increases when moving from trying to sensing. Therefore, our provided instructions, however open-ended, may have prompted participants to try numerous different mental activities, whereas simply “feeling” could have been more effective.

However, results indicate that it is possible for participants to engage with an affective BCI by increasing FAA while experiencing a complex, experimental story environment with imagination-based instructions. It is possible that only those who need less training can be successful with NF interaction with the current system. Despite lower NF success rates than that of Aranyi et al. (2016), participants indicated higher enjoyment than frustration in self-reported data. The role of strategy type (direct or indirect) in FAA NF success is not clear in the present study, and should be investigated in future work. Our study contributes to the goal of making BCI experiences more user-friendly, enjoyable and motivating, while investigating experimental storytelling techniques with which to engage participants during affective BCI interaction.

### 5.1. Limitations and future directions

Our study is limited in a number of different ways. First, we did not collect self-reported data concerning participants' current mood, anxiety, or depression symptoms. Our sample was also very homogeneous: undergraduate students from our department. Ethnic data was not collected. One participant reported currently undergoing psychiatric treatment. Additionally, participants were not asked to refrain from caffeine, cigarettes, alcohol or drugs within 12 h of the experiment. Similar to work by Gapen et al. (2016), this study was intended to be ecologically valid, therefore, its exclusion criteria were limited. However, a positive, effortlessly relaxed mental state may be best for NF interaction in BCIs (Friedrich et al., 2014), therefore, negative moods may have contributed to lack of success seen with our system. Collecting self-reported mood ratings before and after the NF protocol (Zotev et al., 2020) would additionally allow us to learn more about the participant experience. Considering that the participant's motivation, locus of control as well as empathy levels can play a role in BCI performance (Friedrich et al., 2014), a greater focus on participant characteristics would inform both NF success rates with experimental BCIs, and design techniques for future BCI experiences.

In future work, we would like to explore BCI usage in an environment outside the university or departmental setting, with the goal of investigating a more diverse group of participants. A replication of this study using a medical-grade EEG device may also allow us to learn more about participants' success rate during NF interaction, although likely within a less comfortable, quick, and convenient experience and time frame for participants.

Although designed to stimulate the imagination, many factors of the experimental story BCI may have counteracted NF success for certain participants. Although not mentioned as confusing or disorienting to participants, it is possible that the manipulation of spatial logic throughout story blocks contributed to a lower NF success rate through a general sense of displacement. Complex stimuli, such as the developed multi-component experimental story interface, may have effects on the learner (participants) which are not yet understood (Enriquez-Geppert et al., 2017). A follow up study could examine experimental storytelling components individually, in order to understand the influence of each component. A few examples of storytelling components to investigate include story block order, depth of spatio-temporal discontinuity, distance between two characters, vividness of environmental objects, and realism of 3d models among a plethora of other possibilities.

Individuals clearly differ in their preference for story genre and style. In future work, we believe an investigation of learner (participant) preferences for story ambiguity will be central in understanding the influence of experimental storytelling in BCI experiences. Future work could also explore guiding participant attention more directly within stories of varying ambiguity. Lastly, examining NF success with blocks of longer duration could allow participants who need more training to be successful. Instead of 30 s, a longer NF block duration, such as 60 s (Dehghani et al., 2020) may be necessary for some participants to increase FAA within the block.

## Data availability statement

The datasets presented in this study can be found in online repositories. The names of the repository/repositories and accession number(s) can be found at: https://osf.io/uqgp8/?view_only=f6df8369f68c47c59ea0f21308ddba50.

## Ethics statement

The studies involving human participants were reviewed and approved by Purdue University Institutional Review Board. The patients/participants provided their written informed consent to participate in this study. Written informed consent was obtained from the individual(s) for the publication of any potentially identifiable images or data included in this article.

## Author contributions

CK developed the concept, Unity environment, and conducted data collection. CK and BC performed EEG processing and analyses and wrote the manuscript. CK, CM, and BC completed statistical analyses and revised the manuscript. CM acquired funding for the study. All authors listed have made a substantial, direct, and intellectual contribution to the work and approved it for publication.

## Conflict of interest

The authors declare that the research was conducted in the absence of any commercial or financial relationships that could be construed as a potential conflict of interest.

## Publisher's note

All claims expressed in this article are solely those of the authors and do not necessarily represent those of their affiliated organizations, or those of the publisher, the editors and the reviewers. Any product that may be evaluated in this article, or claim that may be made by its manufacturer, is not guaranteed or endorsed by the publisher.

## Footnotes

^1^ https://www.emotiv.com/epoc-x/

^2^ https://www.mixamo.com/

## References

[B1] Albu C. (2020). Intimate connections: Alternative communication threads in nina sobell's video performances and installations (1974-82). Camera Obscura 35, 39–75. 10.1215/02705346-8085111

[B2] Allen J. J. Coan J. A. Nazarian M. (2004). Issues and assumptions on the road from raw signals to metrics of frontal EEG asymmetry in emotion. Biol. Psychol. 67, 183–218. 10.1016/j.biopsycho.2004.03.007 15130531

[B3] Aranyi G. Pecune F. Charles F. Pelachaud C. Cavazza M. (2016). Affective interaction with a virtual character through an fnirs brain-computer interface. Front. Comput. Neurosci. 10:70. 10.3389/fncom.2016.00070 27462216 PMC4940367

[B4] Aranyi G. Charles F. Cavazza M. (2015b). Anger-based BCI using FNIRs neurofeedback, in Proceedings of the 28th Annual ACM Symposium on User Interface Software & Technology (Charlotte, NC: ACM), 511–521. 10.1145/2807442.2807447

[B5] Aranyi G. Cavazza M. Charles F. (2015a). Using FNIRs for prefrontal-asymmetry neurofeedback: methods and challenges, in International Workshop on Symbiotic Interaction (Berlin: Springer), 7–20. 10.1007/978-3-319-24917-9_2

[B6] Ben-Israel A. Greville T. N. (2003). Generalized Inverses. New York, NY: Springer-Verlag.

[B7] Benjamini Y. Hochberg Y. (1995). Controlling the false discovery rate: a practical and powerful appoach to multiple testing. J. R. Stat. Soc. B 57, 289–300. 10.1111/j.2517-6161.1995.tb02031.x

[B8] Berger A. M. Davelaar E. J. (2018). Frontal alpha oscillations and attentional control: a virtual reality neurofeedback study. Neuroscience 378, 189–197. 10.1016/j.neuroscience.2017.06.007 28642166

[B9] Briesemeister B. B. Tamm S. Heine A. Jacobs A. M. (2013). Approach the good, withdraw from the bad-a review on frontal alpha asymmetry measures in applied psychological research. Psychology 4, 261–267. 10.4236/psych.2013.43A039

[B10] Cattan G. (2021). The use of brain-computer interfaces in games is not ready for the general public. Front. Comput. Sci. 3:628773. 10.3389/fcomp.2021.628773

[B11] Cavazza M. Aranyi G. Charles F. (2017). Bci control of heuristic search algorithms. Front. Neuroinform. 11:6. 10.3389/fninf.2017.00006 28197092 PMC5281622

[B12] Cavazza M. Aranyi G. Charles F. Porteous J. Gilroy S. Klovatch I.. (2014a). Towards empathic neurofeedback for interactive storytelling, in 2014 Workshop on Computational Models of Narrative (Quebec City, QC: Schloss Dagstuhl-Leibniz-Zentrum fuer Informatik).

[B13] Cavazza M. Charles F. Aranyi G. Porteous J. Gilroy S. W. Raz G.. (2014b). Towards emotional regulation through neurofeedback, in Proceedings of the 5th Augmented Human International Conference (Kobe), 1–8. 10.1145/2582051.2582093

[B14] Charles F. Martins C. D. C. Cavazza M. (2020). Prefrontal asymmetry bci neurofeedback datasets. Front. Neurosci. 14:601402. 10.3389/fnins.2020.601402 33390885 PMC7775574

[B15] Cohen A. Keynan J. N. Jackont G. Green N. Rashap I. Shani O.. (2016). Multi-modal virtual scenario enhances neurofeedback learning. Front. Robot. AI 3:52. 10.3389/frobt.2016.00052

[B16] Cohen S. S. Henin S. Parra L. C. (2017). Engaging narratives evoke similar neural activity and lead to similar time perception. Sci. Rep. 7, 1–10. 10.1038/s41598-017-04402-4 28676688 PMC5496904

[B17] Davelaar E. J. Barnby J. M. Almasi S. Eatough V. (2018). Differential subjective experiences in learners and non-learners in frontal alpha neurofeedback: piloting a mixed-method approach. Front. Hum. Neurosci. 2018:402. 10.3389/fnhum.2018.00402 30405374 PMC6206258

[B18] David O. A. Predatu R. Maffei A. (2021). Ret Hink online video game for children and adolescents: effects on state anxiety and frontal alpha asymmetry. Int. J. Cogn. Ther. 14, 399–416. 10.1007/s41811-020-00077-4

[B19] Dehghani A. Soltanian-Zadeh H. Hossein-Zadeh G.-A. (2020). Global data-driven analysis of brain connectivity during emotion regulation by electroencephalography neurofeedback. Brain Connect. 10, 302–315. 10.1089/brain.2019.0734 32458692

[B20] Delorme A. Makeig S. (2004). Eeglab: an open source toolbox for analysis of single-trial EEG dynamics including independent component analysis. J. Neurosci. Methods 134, 9–21. 10.1016/j.jneumeth.2003.10.009 15102499

[B21] Dmochowski J. P. Sajda P. Dias J. Parra L. C. (2012). Correlated components of ongoing EEG point to emotionally laden attention-a possible marker of engagement? Front. Hum. Neurosci. 6:112. 10.3389/fnhum.2012.00112 22623915 PMC3353265

[B22] Enriquez-Geppert S. Huster R. J. Herrmann C. S. (2017). EEG-neurofeedback as a tool to modulate cognition and behavior: a review tutorial. Front. Hum. Neurosci. 11:51. 10.3389/fnhum.2017.00051 28275344 PMC5319996

[B23] Fischer R. A. (1971). The Design of Experiments, 9th Edn. New York, NY: Macmillan Publishing Co.

[B24] Friedrich E. V. Wood G. Scherer R. Neuper C. (2014). Mind over brain, brain over mind: cognitive causes and consequences of controlling brain activity. Front. Hum. Neurosci. 8:348. 10.3389/fnhum.2014.00348 24904384 PMC4034699

[B25] Gapen M. van der Kolk B. A. Hamlin E. Hirshberg L. Suvak M. Spinazzola J. (2016). A pilot study of neurofeedback for chronic PTSD. Appl. Psychophysiol. Biofeedb. 41, 251–261. 10.1007/s10484-015-9326-5 26782083

[B26] Gilroy S. W. Porteous J. Charles F. Cavazza M. Soreq E. Raz G.. (2013). A brain-computer interface to a plan-based narrative, in Twenty-Third International Joint Conference on Artificial Intelligence (Beijing).

[B27] Gonzalez-Franco M. Egan Z. Peachey M. Antley A. Randhavane T. Panda P.. (2020). Movebox: democratizing MOCAP for the microsoft rocketbox avatar library, in 2020 IEEE International Conference on Artificial Intelligence and Virtual Reality (AIVR) (Urecht: IEEE), 91–98. 10.1109/AIVR50618.2020.00026

[B28] Gruzelier J. Inoue A. Smart R. Steed A. Steffert T. (2010). Acting performance and flow state enhanced with sensory-motor rhythm neurofeedback comparing ecologically valid immersive VR and training screen scenarios. Neurosci. Lett. 480, 112–116. 10.1016/j.neulet.2010.06.019 20542087

[B29] Harmon-Jones E. Gable P. A. (2018). On the role of asymmetric frontal cortical activity in approach and withdrawal motivation: an updated review of the evidence. Psychophysiology 55:e12879. 10.1111/psyp.12879 28459501

[B30] Hart S. G. (2006). NASA-task load index (NASA-TLX); 20 years later, in Proceedings of the Human Factors and Ergonomics Society Annual Meeting, Vol. 50 (Los Angeles, CA: Sage publications), 904–908. 10.1177/154193120605000909

[B31] Hasson U. Landesman O. Knappmeyer B. Vallines I. Rubin N. Heeger D. J. (2008). Neurocinematics: the neuroscience of film. Projections 2, 1–26. 10.3167/proj.2008.020102

[B32] Jensen C. B. F. Petersen M. K. Larsen J. E. Stopczynski A. Stahlhut C. Ivanova M. G.. (2013). Spatio temporal media components for neurofeedback, in 2013 IEEE International Conference on Multimedia and Expo Workshops (ICMEW) (San Jose, CA: IEEE), 1–6. 10.1109/ICMEW.2013.6618362

[B33] Johnston S. Linden D. E. J. Healy D. Goebel R. Habes I. Boehm S. (2011). Upregulation of emotion areas through neurofeedback with a focus on positive mood. Cogn. Affect. Behav. Neurosci. 11, 44–51. 10.3758/s13415-010-0010-1 21264651

[B34] Kelley N. J. Hortensius R. Schutter D. J. Harmon-Jones E. (2017). The relationship of approach/avoidance motivation and asymmetric frontal cortical activity: a review of studies manipulating frontal asymmetry. Int. J. Psychophysiol. 119, 19–30. 10.1016/j.ijpsycho.2017.03.001 28288803

[B35] Kerous B. Skola F. Liarokapis F. (2018). EEG-based BCI and video games: a progress report. Virt. Real. 22, 119–135. 10.1007/s10055-017-0328-x

[B36] Kisker J. Lange L. Flinkenflügel K. Kaup M. Labersweiler N. Tetenborg F.. (2021). Authentic fear responses in virtual reality: a mobile EEG study on affective, behavioral and electrophysiological correlates of fear. Front. Virt. Real. 2:716318. 10.3389/frvir.2021.716318

[B37] Koenitz H. (2017). Beyond ‘walking simulators'-games as the narrative avant-garde, in Digital Games Research Association Conference (Melbourne, VIC).

[B38] Kosmyna N. Lécuyer A. (2017). Designing guiding systems for brain-computer interfaces. Front. Hum. Neurosci. 11:396. 10.3389/fnhum.2017.00396 28824400 PMC5535189

[B39] Kuper N. Käckenmester W. Wacker J. Fajkowska M. (2019). Resting frontal EEG asymmetry and personality traits: a meta-analysis. Eur. J. Pers. 33, 154–175. 10.1002/per.2197

[B40] Lackner N. Unterrainer H.-F. Skliris D. Shaheen S. Dunitz-Scheer M. Wood G.. (2016). EEG neurofeedback effects in the treatment of adolescent anorexia nervosa. Eating Disord. 24, 354–374. 10.1080/10640266.2016.1160705 27027700

[B41] Le Groux S. Manzolli J. Verschure P. F. Sanchez M. Luvizotto A. Mura A.. (2010). Disembodied and collaborative musical interaction in the multimodal brain orchestra, in NIME (Sydney, NSW), 309–314.

[B42] Le T. P. Lucas H. D. Schwartz E. K. Mitchell K. R. Cohen A. S. (2020). Frontal alpha asymmetry in schizotypy: electrophysiological evidence for motivational dysfunction. Cogn. Neuropsychiatry 25, 371–386. 10.1080/13546805.2020.1813096 32873177

[B43] Mennella R. Patron E. Palomba D. (2017). Frontal alpha asymmetry neurofeedback for the reduction of negative affect and anxiety. Behav. Res. Ther. 92, 32–40. 10.1016/j.brat.2017.02.002 28236680

[B44] Mori. (2003). Wave UFO, Vol. 3. Available online at: https://www.publicartfund.org/exhibitions/view/wave-ufo/ (accessed June 10, 2022).

[B45] Nijholt A. (2015). Competing and collaborating brains: multi-brain computer interfacing, in Brain-Computer Interfaces, eds Hassanien A. E. Azar A. T. (New York, NY: Springer), 313–335. 10.1007/978-3-319-10978-7_12

[B46] Parra L. C. Spence C. D. Gerson A. D. Sajda P. (2005). Recipes for the linear analysis of EEG. NeuroImage 28, 326–341. 10.1016/j.neuroimage.2005.05.032 16084117

[B47] Peeters F. Ronner J. Bodar L. van Os J. Lousberg R. (2014). Validation of a neurofeedback paradigm: manipulating frontal EEG alpha-activity and its impact on mood. Int. J. Psychophysiol. 93, 116–120. 10.1016/j.ijpsycho.2013.06.010 23773999

[B48] Pike M. Wilson M. L. Benford S. Ramchurn R. (2016). # scanners: a Bci enhanced cinematic experience, in Proceedings of the 2016 CHI Conference Extended Abstracts on Human Factors in Computing Systems (San Jose, CA), 293–296. 10.1145/2851581.2889468

[B49] Quaedflieg C. W. Smulders F. T. Meyer T. Peeters F. Merckelbach H. Smeets T. (2016). The validity of individual frontal alpha asymmetry EEG neurofeedback. Soc. Cogn. Affect. Neurosci. 11, 33–43. 10.1093/scan/nsv090 26163671 PMC4692315

[B50] Robinson R. Wiley K. Rezaeivahdati A. Klarkowski M. Mandryk R. L. (2020). ‘Let's get physiological, physiological!' A systematic review of affective gaming, in Proceedings of the Annual Symposium on Computer-Human Interaction in Play, 132–147. 10.1145/3410404.3414227

[B51] Rosenfeld J. P. Cha G. Blair T. Gotlib I. H. (1995). Operant (biofeedback) control of left-right frontal alpha power differences: potential neurotherapy for affective disorders. Biofeedb. Self-Regul. 20, 241–258. 10.1007/BF01474516 7495918

[B52] Saha S. Mamun K. A. Ahmed K. I. U. Mostafa R. Naik G. R. Darvishi S.. (2021). Progress in brain computer interface: challenges and potentials. Front. Syst. Neurosci. 15:4. 10.3389/fnsys.2021.578875 33716680 PMC7947348

[B53] Sitaram R. Ros T. Stoeckel L. Haller S. Scharnowski F. Lewis-Peacock J.. (2017). Closed-loop brain training: the science of neurofeedback. Nat. Rev. Neurosci. 18, 86–100. 10.1038/nrn.2016.164 28003656

[B54] Smith E. E. Reznik S. J. Stewart J. L. Allen J. J. (2017). Assessing and conceptualizing frontal EEG asymmetry: an updated primer on recording, processing, analyzing, and interpreting frontal alpha asymmetry. Int. J. Psychophysiol. 111, 98–114. 10.1016/j.ijpsycho.2016.11.005 27865882 PMC6449497

[B55] Szczelkun S. (2018). SENSE THINK ACT: A Collection Of Exercises to Experience Total Human Ability, Vol. 1. London: Routine Art Co.

[B56] Taberham P. (2018). Lessons in Perception: The Avant-Garde Filmmaker as Practical Psychologist. New York, NY: Berghahn Books. 10.2307/j.ctv3znzvc

[B57] Templeton G. F. (2011). A two-step approach for transforming continuous variables to normal: implications and recommendations for is research. Commun. Assoc. Inform. Syst. 28:4. 10.17705/1CAIS.02804

[B58] Wadeson A. Nijholt A. Nam C. S. (2015). Artistic brain-computer interfaces: state-of-the-art control mechanisms. Brain Comput. Interfaces 2, 70–75. 10.1080/2326263X.2015.1103155

[B59] Zioga P. Pollick F. Ma M. Chapman P. Stefanov K. (2018). ‘Enheduanna a manifesto of falling' live brain-computer cinema performance: performer and audience participation, cognition and emotional engagement using multi-brain BCI interaction. Front. Neurosci. 12:191. 10.3389/fnins.2018.00191 29666566 PMC5891608

[B60] Zotev V. Mayeli A. Misaki M. Bodurka J. (2020). Emotion self-regulation training in major depressive disorder using simultaneous real-time fMRI and EEG neurofeedback. NeuroImage 27:102331. 10.1016/j.nicl.2020.102331 32623140 PMC7334611

